# Hybrid membranes for the production of blood contacting surfaces: physicochemical, structural and biomechanical characterization

**DOI:** 10.1186/s40824-021-00227-5

**Published:** 2021-08-10

**Authors:** Martina Todesco, Carlo Zardin, Laura Iop, Tiziana Palmosi, Pietro Capaldo, Filippo Romanato, Gino Gerosa, Andrea Bagno

**Affiliations:** 1 Department of Industrial Engineering, University of Padua, via Marzolo 9, 35131 Padova, Italy; 2 L.i.f.e.L.a.b. Program, Consorzio per la Ricerca Sanitaria (CORIS), Veneto Region, Via Giustiniani 2, 35128 Padova, Italy; 3 Department of Cardiac, Thoracic Vascular Sciences and Public Health, University of Padova, via Giustiniani 2, 35128 Padova, Italy; 4 Department of Physics and Astronomy ‘G. Galilei’, University of Padova, via Marzolo 8, 35131 Padova, Italy; 5 CNR-INFM TASC IOM National Laboratory, S.S. 14 Km 163.5, 34012 Basovizza, Trieste, Italy

**Keywords:** Porcine pericardium, Decellularization, Hybrid membrane, Blood contacting surface, Total artificial heart.

## Abstract

**Background:**

Due to the shortage of organs’ donors that limits biological heart transplantations, mechanical circulatory supports can be implanted in case of refractory end-stage heart failure to replace partially (Ventricular Assist Device, VAD) or completely (Total Artificial Heart, TAH) the cardiac function. The hemocompatibility of mechanical circulatory supports is a fundamental issue that has not yet been fully matched; it mostly depends on the nature of blood-contacting surfaces.

**Methods:**

In order to obtain hemocompatible materials, a pool of hybrid membranes was fabricated by coupling a synthetic polymer (polycarbonate urethane, commercially available in two formulations) with a decellularized biological tissue (porcine pericardium). To test their potential suitability as candidate materials for realizing the blood-contacting surfaces of a novel artificial heart, hybrid membranes have been preliminarily characterized in terms of physicochemical, structural and mechanical properties.

**Results:**

Our results ascertained that the hybrid membranes are properly stratified, thus allowing to expose their biological side to blood and their polymeric surface to the actuation system of the intended device. From the biomechanical point of view, the hybrid membranes can withstand deformations up to more than 70 % and stresses up to around 8 MPa.

**Conclusions:**

The hybrid membranes are suitable for the construction of the ventricular chambers of innovative mechanical circulatory support devices.

## Background

Heart failure (HF) is a clinical condition due to a severe impairment of heart function. The synergistic effect of population’s aging and increased patients’ survival after myocardial infarction is causing a dramatic growth of HF prevalence, i.e. 46 % from 2012 to 2030 [1].

Nowadays, the optimal therapeutic solution for refractory end-stage HF is represented by cardiac transplantation, which is limited by organs’ shortage [2]. The quest for alternative therapeutic treatments stimulated the development of mechanical circulatory supports (MCSs): ventricular-assist devices (VADs) successfully support one ventricle, whereas total artificial hearts (TAHs) replace both ventricles. TAH implantation now represents a suitable option for patients requiring biventricular mechanical circulatory support either as bridge to transplant (BTT) or destination therapy (DT) [2, 3].

The experimental use of TAHs dates back to 1937 when Vladimir Demikhov implanted one of the earliest mechanical cardiac substitutes in a dog replacing its heart’s function for as long as 51 h [4]. In 1957 Akutsu and Kolff implanted another pioneering device in a dog to support circulation for one hour and a half [5]. Twelve years later, Cooley and Liotta performed the first TAH implantation in humans: they used the so-called “Liotta Heart” and the patient (a 47-year-old man) was bridged to transplantation after 64 h of support [6].

Over the years, different TAH prototypes were designed, developed and implanted. Up to date, there is only one FDA approved and CE marked device: the Syncardia CardioWest TAH (Tucson, Arizona, USA) [7]. A second TAH, the Carmat (Carmat, Velizy-Villacoublay, France), has received the CE mark on December 2020 [8, 9].

Several drawbacks are still limiting TAHs’ implantation, for example dimensions, weight, actuation, and power supply. Moreover, calcification and infections can cause the device to fail [2] and the lack of full blood-compatibility can - directly and indirectly - result in hemorrhages, hemolysis, thrombosis, and thromboembolism. Many of these drawbacks depend on blood–material interactions that are not easily predictable. The only fully blood-compatible surface is the healthy endothelium that internally covers blood and lymphatic vessels. Therefore, strategies for increasing blood-compatibility were originally based on the formation of an endothelial layer or a pseudo-neointima (PNI) [2]. Other approaches have been recently exploited to get biocompatible, durable, and anti-thrombogenic materials: (i) a combination of hyaluronic acid (HA) with LLPDE (linear low density polyethylene) [10]; (ii) a biostable and biocompatible polymer poly(styrene-block-isobutylene-block-styrene) (SIBS) reinforced with a polyethylene terephthalate (PET) fabric [11]; (iii) a nanocomposite polymer consisting of a hard crystalline segment and soft elastomeric segments with polyhedral oligomericsilsesquioxanes (POSS) nanoparticles attached to the backbone of poly(carbonate-urea) urethane [12]. Indeed, all these materials have been used for the production of polymer-based prosthetic heart valves.

The present work aims at producing and typifying an innovative kind of material, which is intended for the construction of the blood-contacting surfaces of ventricular chambers for a novel TAH. The material is obtained by coupling a synthetic polymer (commercial polycarbonate urethane, available in two different formulations) with a decellularized biological tissue (porcine pericardium).

Synthetic materials have several advantages: particularly, their physicochemical and mechanical properties are likely addressed for the intended application with high reproducibility. Moreover, polymers are easy to be shaped and assure great mechanical resistance even upon cyclic loads. Unfortunately, they cannot assure adequate biocompatibility and, especially, blood-compatibility: these aspects have to be verified each and every time.

Decellularized biological tissues may provide high biocompatibility, but their long-term mechanical resistance is often unsatisfactory: decellularization itself can severely affect tissue stability with the risk of in vivo degradation. A recent review on the preparation of decellularized tissues highlights pros and cons of the existing decellularization procedures, focusing the importance of maintaining adequate compositional, structural and mechanical features [13].

To overcome limitations and combine advantages of both synthetic polymers and decellularized tissues, a hybrid strategy was proposed to create a new class of materials, whose properties are customizable. This strategy has been applied in the Carmat TAH: the membranes separating blood from the actuation fluid (silicone oil) are of hybrid nature. The blood-contacting side of the membranes is made of bovine pericardium; the other side is composed of a polycarbonate urethane [14]. In this case, pericardium is chemically treated with glutaraldehyde (GA) to achieve long-term tolerance and hemocompatibility; indeed, GA is known to cause cytotoxic effects and calcification [15, 16]. For these reasons, in the present research decellularized pericardium was preferred. After removing cellular and nuclear materials, decellularized tissues lose their immunogenic properties and are prone to be repopulated by circulating cells [17]. Therefore, the hybrid membranes are expected to withstand the mechanical loads due to cyclic actuation into the intended pulsatile ventricular chambers thanks to the polymeric layer, but also to allow re-endothelization thanks to the decellularized tissue.

In this paper, the hybrid membranes were characterized with regard to composition, structure and mechanical properties, considering their potential application.

## Materials and methods

### Pericardia preparation and decellularization procedure

Fresh pericardia of healthy animals (Duroc pigs, 9–14-months old, weight between 140 and 170 kg) were supplied from local abattoirs and transferred to the laboratory in sterile condition in saline solution (0.9 % NaCl, Sigma-Aldrich, Saint Louis, MO, USA). Within 2 h after animal death, pericardium was dissected free from its attachment at the base of the heart around great vessels; it was cleaned by removing retrosternal fat and surrounding connective tissues and shortly rinsed in cold saline solution. For the fabrication of hybrid membranes, samples of porcine pericardium withdrawn from the anterior right ventricular region were used.

Tissues were treated according to the TRICOL procedure (based on Triton X-100 and sodium colate), which was already used by our group to decellularize aortic valves [18] and pericardia [17]. The procedure was carried out under constant agitation at 4 °C and following 8 h-long cycles. The inactivation of cell proteases was followed by alternated hypo- and hypertonic solutions, combined with decreasing concentrations of Triton X-100 (1–0.1 %, Sigma-Aldrich). Cellular components were extracted from tissue by using 10 mM bile salt anionic surfactant sodium cholate (Sigma-Aldrich). Extractions were performed in a degassed solution containing 10 mM sodium ascorbate (Sigma-Aldrich) and 5 mM EDTA (ethylenediaminetetraacetic acid, Sigma-Aldrich) under nitrogen atmosphere.

Residual nucleic acids were digested using 1500 U cm^− 2^ non-specific endonucleases (Benzonase, Sigma-Aldrich) that degrades single- and double-stranded DNA and RNA. A solution with equilibration buffer, 50 mM tris HCl and 1 mM MgCl_2_, both supplied by Sigma Aldrich, and Benzonase was maintained for 48 h at 37 °C.

After decellularization, pericardial samples were stored at 4 °C in antibiotic and antimycotic solution (3 % penicillin-streptomycin (Sigma-Aldrich) and 0.25 % Amphotericin B (Carlo Erba, Milan, Italy)).

In the following sections, native and decellularized porcine pericardia will be indicated as NPP and DPP, respectively.

### DNA content and histological evaluation

DNA extraction, histological evaluation and immunofluorescence staining were performed on NPP and DPP samples to evaluate the effectiveness of TRICOL decellularization and analyze extracellular matrix (ECM) composition. NPP was used as control to evaluate changes caused by decellularization.

Total DNA content was quantified by means of DNeasy Blood & Tissue Kit (Quiagen, Valencia, CA, USA), according to the recommended protocol. NPP and DPP samples (n = 9) were dried with filter paper, weighted, minced and rehydrated using the solutions supplied. DNA concentration was determined with NanoDrop 2000 spectrophotometer (Thermo Fisher Scientific, Waltham, MA, USA): each extracted DNA sample (1 µL) was loaded onto the instrument and the absorbance was measured at 260 nm. DNA concentrations, expressed in micrograms per milligrams of dry tissue, were calculated after normalization per dry tissue weight.

Histological analyses were performed on NPP and DPP. Samples were dried with filter paper, embedded in Optimum Cutting Temperature (OCT) compound (Tissue-Tek, Alphen Aan den Rijn, Netherlands) and snap freezing in liquid nitrogen. Sample Sec. (8 μm) were fixed in 2 % paraformaldehyde (PFA) for 5 min and stained with hematoxylin/eosin (H&E) supplied by Bioptica (Milan, Italy). Tissues were assessed with EVOS XL Core Cell Imaging System (Thermo Fisher Scientific, Waltham, MA, USA).

Immunofluorescence staining was performed as already described in [17].

### Hybrid membranes fabrication

**Fig. 1:**
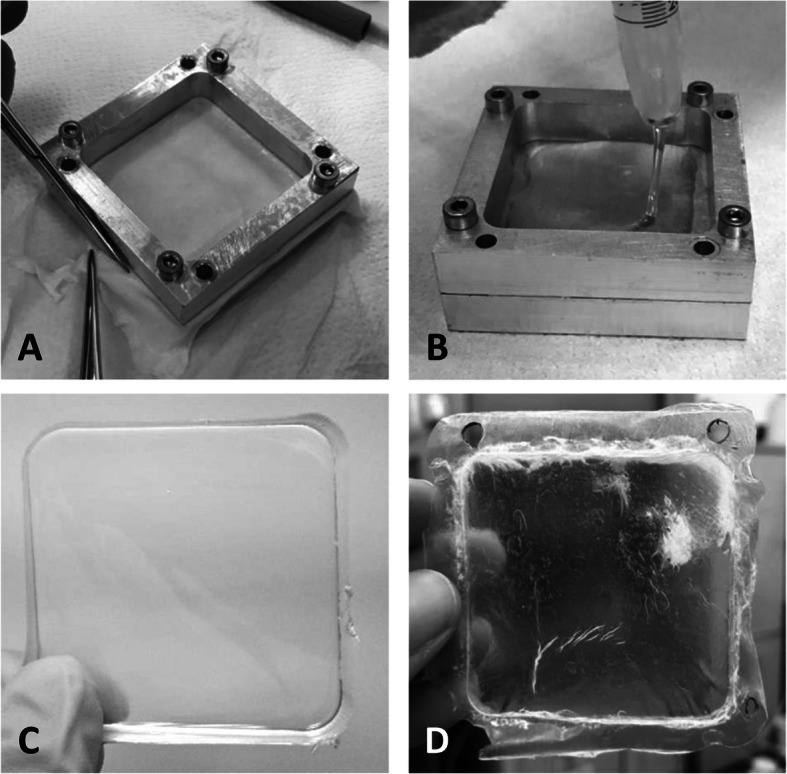
The steps for obtaining the hybrid membrane. **A**) Porcine pericardial sample is placed into the metallic frame on the serosa side and fixed. **B**) The polymeric solution is gently poured over the fibrosa side of the pericardial sample. **C**) The polymeric membrane as it appears after drying. **D**) The hybrid membrane obtained by coupling porcine pericardium and Chronoflex AR

Hybrid membranes were produced by solution casting and solvent evaporation [19]. Decellularized tissue samples were washed twice for 30 min in milliQ water, then gently dried with filter paper. Afterwards, biological samples were placed on the serosa side and fastened into a customized 50 × 50 mm^2^ aluminum frame (Fig. 1). Over the other side (fibrosa) a thin layer (5 mL) of commercial polycarbonate urethane (PCU) solution was gently poured (ChronoFlex AR and ChronoFlex AR-LT, CF, AdvanSource Biomaterials, Wilmington, MA, US). The hybrid membranes were dried for 24 h at 40 °C into a vacuum oven under aspiration (Raypa, Barcelona, Spain).

Commercial PCUs are composed of aromatic diisocyanates, such as methylene diisocyanate (MDI), polycarbonate diols, 1,3 diaminocyclohexane, and ethylene diamine (EDA) [20]. The commercial product identified as ChronoFlex AR-LT differs from ChronoFlex AR since it contains silica microparticles that make the polymer less tacky. The choice for these commercial products is justified by their bio- and hemocompatibility and their availability in liquid: they are supplied as 22 % (w/v) solutions in N,N-dimethylacetamide (DMAc). Therefore, they are suitable for molding, casting and dip-coating fabrication techniques. Both products are expected to resist environmental stress cracking and to exhibit high flexural endurance in applications such as blood contacting surfaces of artificial hearts [21].

Polymeric membranes were obtained by dispending polycarbonate urethane solutions into the metallic frame and drying them under vacuum for 24 h at 80° C. After complete drying and cooling, polymeric membranes were carefully detached from the frame.

In the following sections, polymeric membranes will be indicated as AR and AR-LT, depending on the polymer formulation; hybrid membranes will be indicated as DPP-AR and DPP-ARLT, depending on the polymer formulation.

### Thickness measurements and mechanical tests

**Fig. 2:**
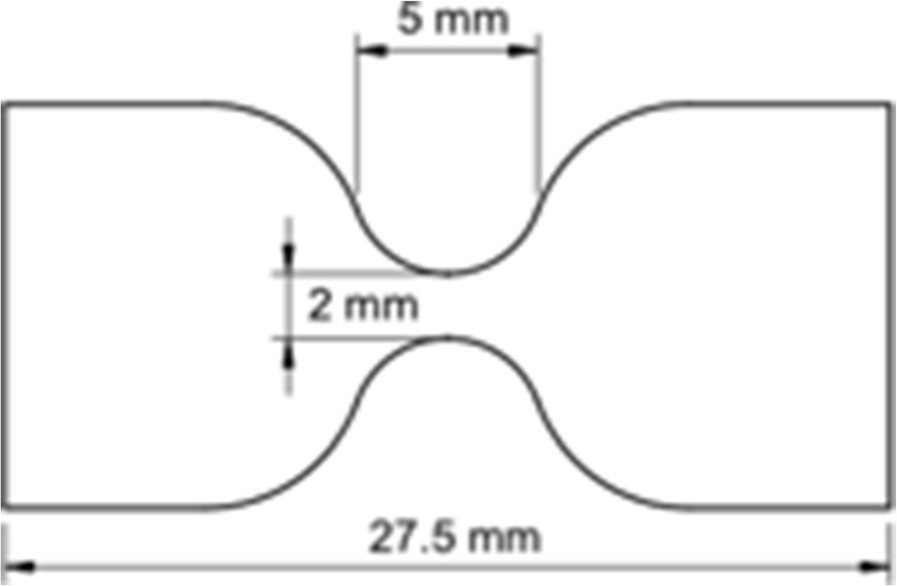
Dog bone shaped specimen. Shape and dimension (mm) of the dog bone shaped specimens used for the mechanical tests

For the mechanical assessment, all samples were cut into dog bone shaped specimens with a gauge length of 5 mm and 2 mm width (Fig. 2) by means of an in-house designed cutter. The choice for this sample shape is based on the ASTM D1708-13 standard concerning small-size tissues.

Samples thickness was measured using a Mitutoyo digital caliber (model ID-C112XB, Aurora, Illinois, USA) sandwiching them between two glass slides, whose thickness is then subtracted. Thickness mean values and standard deviations were calculated (n = 9, six measures for each sample) (Table 1).

**Table 1:** Sample thickness (mm ± standard deviation)

Sample	Thickness [mm]
NPP	0.15 ± 0.03
DPP	0.25 ± 0.05
DPP-AR	0.38 ± 0.10
DPP-ARLT	0.50 ± 0.10

Samples were biomechanically assessed by uniaxial tensile loading tests, which were performed with a custom-made apparatus (IRS, Padova, Italy). The system was equipped with four linear actuators and four loading cells (50 N). Uniaxial tests were performed using two actuators and two cells at room temperature; samples were continuously wetted with 0.9 % NaCl solution to prevent dehydration. Samples were preloaded up to 0.1 N, then elongated (elongation rate 1 mm/s) to rupture for measuring the Ultimate Tensile Strength (UTS) and the Failure Strain (FS). The elastic modulus (E) was calculated as the slope of the stress-strain curves in the linear region. Engineering stress σ (MPa) was defined as the tensile force measured by the loading cells (Newton) divided by the original cross-sectional area of the sample; the strain ε (%) was defined as the ratio between the grip displacement and the gauge length.

### Physicochemical characterization

Biological tissues, polymeric membranes and hybrid membranes were individually characterized by means of the analytical techniques that are briefly described herewith.

#### Differential Scanning Calorimetry (DSC)

Small samples of the polymeric membranes (ChronoFlex AR: 19.4 mg; ChronoFlex AR-LT: 19.2 mg) and samples of the hybrid membranes (DPP-AR: 19 mg; DPP-ARLT: 17.7 mg) were analysed by means of the DSC Q200 calorimeter (TA Instruments, New Castel, Delaware, USA). Specimens were placed into sealed aluminium cups, then heated up to 200° C, cooled down to -70° C, and heated again up to 200° C (heating/cooling rate of 20 K/min). Heating/cooling cycles allowed neglecting the effects of thermal and mechanical history on samples. Transition temperatures were measured during the ramp from − 70 to 200° C.

#### Thermo-Gravimetric Analysis (TGA)

The thermogravimetric analyses were carried out with the SDT Q600 apparatus (TA Instruments, New Castel, Delaware, USA) in oxidizing atmosphere (air) to ascertain the amount of silica microparticles added to the ChronoFlex AR-LT. Polymer thermal degradation was provoked by increasing temperature up to 1000° C with a rate of 20 K/min. The weight of ChronoFlex AR-LT sample was 19.3 mg.

#### Fourier Transform Infrared spectroscopy Attenuated Total Reflection (FTIR-ATR)

These analyses were performed by means of the Nicolet iS-50 spectrometer (Thermo Fisher Scientific, Waltham, Massachusetts, USA) in the attenuated total reflectance (ATR) mode on the polymeric membranes, on the biological samples and on the pericardial side of the hybrid membrane. IR spectra were collected in the 4000 − 500 cm^− 1^ range to characterize the composition of each material. Thus, spectra were overlapped to compare the composition of the investigated materials.

### SEM and two-photon microscopy

SEM and two-photon microscopy analyses were carried out on hybrid membranes (DPP-AR and DPP-ARLT). Before SEM analysis, all specimens were vacuum metalized with Rh-Au (2–3 nm thickness). SEM images were acquired with a LEO XB-1540 dual beam FIB-SEM (Zeiss, Oberkochen, Germany), an instrument with the potential for nanometer resolution. The acquisition parameters included high vacuum mode, accelerating voltage of 5 kV, working distance of ∼5 mm, and ion beam current of ∼60 pA for precision milling. A circular backscatter detector and a magnification of 124x were used.

Multiphoton analysis was performed by the custom-built multimodal microscope described in [22]. Second harmonic generation (SHG) was detected and the chosen excitation was 800 nm. Images were acquired with a fixed resolution of 1024 × 1024 pixels and accumulation of 120 frames, performed with a pixel dwell time of 0.14 μm. After acquisition, images were processed with Fiji software, an open source platform for biological image analysis.

### Data analysis and statistical test

Data were averaged over 9/12 samples for each experimental condition. Significant differences were detected by Student’s t-test and ANOVA test (p < 0.05).

## Results

### Biological characterization

**Fig. 3:**
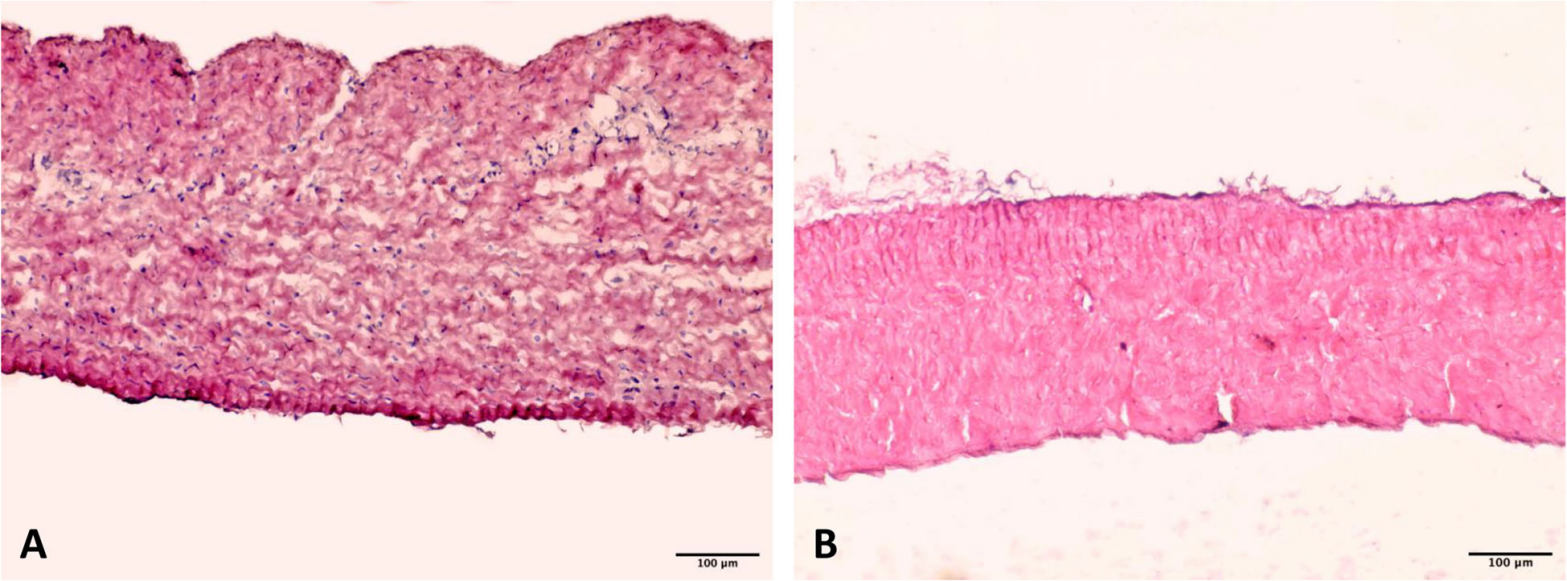
Histological evaluation before and after decellularization. H&E staining highlights the presence of many nuclei in NPP (**A**), while they are not present in DPP (**B**). Extracellular matrix appears preserved after decellularization. Scale bar = 100 µm

The amounts of DNA in NPP and DPP samples were 500.7 ± 85.3 and 46.1 ± 3.6 ng⋅mg^− 1^, respectively. TRICOL decellularization caused a significant decrease of the DNA amount (− 90.8 %) with respect to the native tissue (p = 0.0006). Histological evaluations confirmed that cells were effectively removed, while collagen bundles even showed their characteristic wavy patterns in both DPP and NPP samples (Fig. 3). Immunofluorescence staining also revealed that collagen and elastin architecture was preserved after decellularization (data nor shown).

### Biomechanical characterization

With regard to samples thickness, decellularization caused a slight increase from 0.15 ± 0.03 to 0.25 ± 0.05 mm. As to the hybrid membranes, DPP-ARLT samples were thicker (0.50 ± 0.10 mm) than DPP-AR ones (0.38 ± 0.10 mm).

The results obtained from the tensile tests on NPP, DPP and on the hybrid membranes (DPP-AR and DPP-ARLT) are summarized in Table 2.

**Table 2:** Mechanical characterization in terms of elastic modulus (E), ultimate tensile strength (UTS) and failure strain (FS). Mean values ± standard deviation are shown

Sample	E [MPa]	UTS [MPa]	FS [%]
NPP	10.57 ± 3.28	13.75 ± 3.41	82.1 ± 21.0
DPP	8.49 ± 4.14	11.96 ± 5.94	76.0 ± 29.3
DPP-AR	8.89 ± 2.85	8.18 ± 2.45	71.9 ± 21.4
DPP-ARLT	7.32 ± 1.20	8.02 ± 3.57	76.6 ± 20.0

A decrease in stiffness appears when NPP (E = 10.57 ± 3.28 MPa) was decellularized (E = 8.49 ± 4.14 MPa) and combined with both polymers: it was more evident for DPP-ARLT than for DPP-AR (7.32 ± 1.20 MPa and 8.89 ± 2.85 MPa, respectively). Correspondingly, UTS values were higher for NPP (13.75 ± 3.41 MPa) compared to the hybrid membranes: 8.18 ± 2.45 MPa for DPP-AR and 8.02 ± 3.57 MPa for DPP-ARLT. FS values followed a similar trend: they decreased after decellularization (from 82.1 ± 21.0 to 76.0 ± 29.3 %). When DPP was coupled with ChronoFlex AR-LT, which contains 9 % microsilica, FS values did not change (76. 0 ± 29.3 % versus 76.6 ± 20.0 %); a decrease of FS values was gained when DPP was coupled with ChronoFlex AR (71.90 ± 21.4 %).

### Physicochemical characterization

#### Differential Scanning Calorimetry (DSC)

**Fig. 4:**
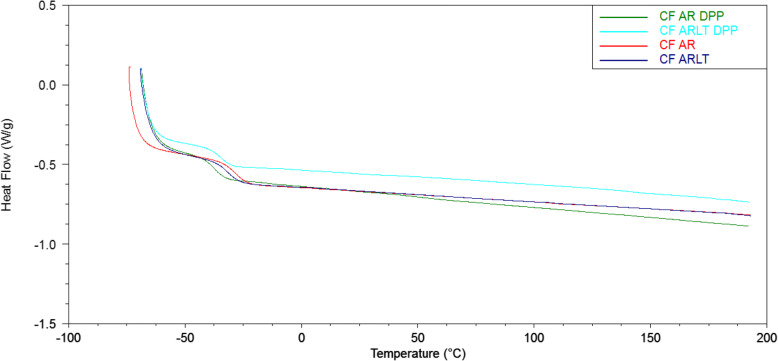
DSC analysis of polymers and hybrid membranes. Glass transition temperatures of Chronoflex AR and AR-LT are -28° C and -31.5 ° C, respectively. Glass transition temperatures of hybrid membranes are -41.49° C and -38.08° C for DPP-AR and DPP-ARLT, respectively

Figure 4 shows DSC thermograms corresponding to Chronoflex AR, Chronoflex AR-LT, DPP-AR and DPP-ARLT. DSC curves show the glass transition temperatures (T_g_): they are − 31.5° C for ChronoFlex AR-LT, and – 28° C for ChronoFlex AR. Hybrid membranes exhibit lower T_g_: -41.49° C for DPP-AR and − 39.08° C for DPP-ARLT. The T_g_ of Chronoflex AR-LT is lower than Chronoflex AR probably because of the presence of silica micro-particles, which entail a higher degree of phase separation between hard and soft segments of the polymer [23]. The presence of the decellularized biological tissue results in a further decrease of the transition temperatures.

#### Thermo-Gravimetric Analysis (TGA)

**Fig. 5:**
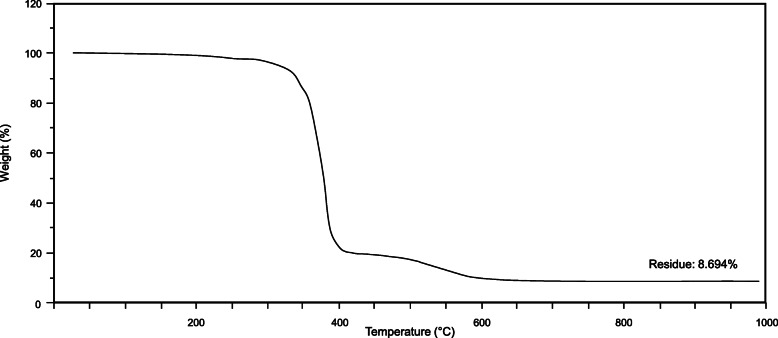
TGA analysis of Chronoflex AR-LT. From the graph the presence of almost 9% inorganic residue (silica micro-particles) is ascertained

TGA confirmed the presence of a residual inorganic amount (~ 9 %) at a temperature higher than 550 °C in the ChronoFlex AR-LT (Fig. 5): this is due to the addition of silica micro-particles, which make the polycarbonate urethane less tacky.

#### Fourier Transform Infrared spectroscopy Attenuated Total Reflection (FTIR-ATR)

**Fig. 6:**
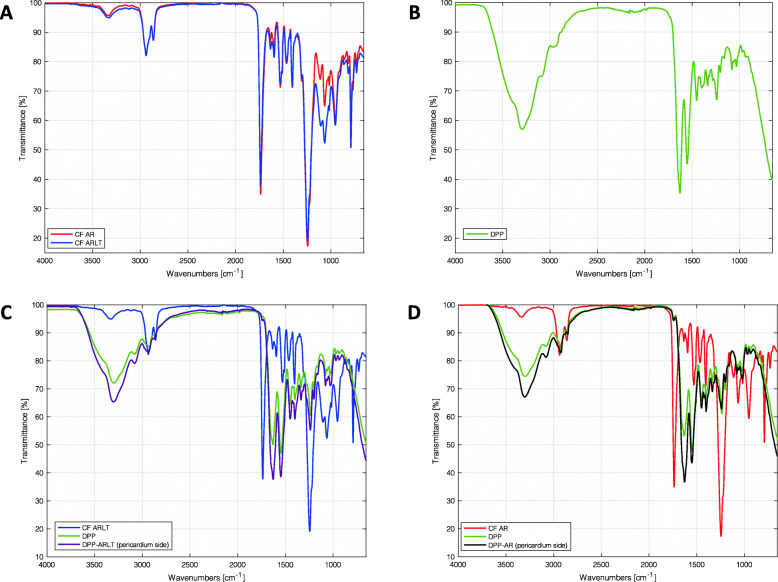
FTIR-ATR spectra.**A**) Chronoflex AR (red line) and Chronoflex AR-LT (blue line); **B**) decellularized porcine pericardium (DPP); **C**) porcine pericardium (green line), ChronoFlex AR-LT (blu line) and hybrid membrane on the pericardium side (violet line); **D**) porcine pericardium (green line), ChronoFlex AR (red line) and hybrid membrane on the pericardium side (violet line)

Chemical compositions of porcine pericardium and polycarbonate urethanes were separately assessed by FTIR-ATR. Polymers spectra were largely overlapped (Fig. 6 A) with the evidence of common peaks at 1737 and 1251 cm^− 1^, typical of urethane and carbonate groups. Peaks around 1251, 956 and 791 cm^− 1^ were marked and assigned to carbonate groups in the soft segment. The peak at 1598 cm^− 1^ suggests that the hard segment of the polymers contains aromatic molecules, while the peak around 1637 cm^− 1^ can be assigned to the presence of diamine (probably used as chain extender) [24]. Signal intensities were different in the interval 1200 − 900 cm^− 1^: this can be due the presence of silica micro-particles in the Chronoflex AR-LT.

FTIR-ATR spectrum of DPP (Fig. 6B) presented a peak between 3000 and 3500 cm^− 1^ due to the hydrogen bond; peaks between 1800 and 900 cm^− 1^ were assigned to collagen, which is the most abundant tissue component. Amide I and amide II bands, classical major bands of this protein [25], absorb between 1510 and 1580 cm^− 1^ and 1600–1700 cm^− 1^ and are primarily associated with the stretching vibrations of C = O in carbonyl groups and N-H bending of amino groups [26, 27].

FTIR-ATR spectra were also acquired on the pericardial side of the hybrid membranes to ascertain that polymers did not diffuse throughout the biological tissue because of the solvent casting procedure. This result is not in agreement with [19] where bovine pericardium was used, indeed. IR penetration depth was about 30 μm and the absence of polymer traces assured that the blood-contacting surface of the membrane exposed the decellularized porcine pericardium only (Fig. 6, C and D).

### Structural characterization

**Fig. 7:**
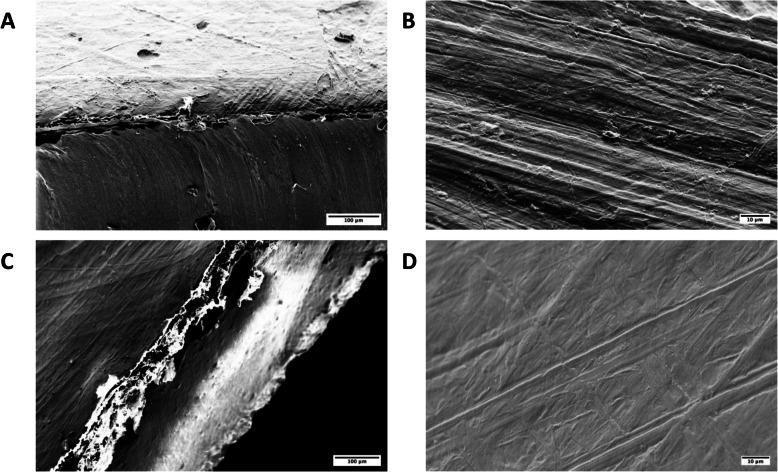
SEM image of the DPP-AR and DPP-ARLT membranes. Cross-sections of DPP-AR and DPP-ARLT membranes are depicted in A) and C): the pericardial layer (top) and the polymer layer (bottom) are clearly visible (scale bar = 100 µm). Surfaces (pericardial side) of DPP-AR and DPP-ARLT membranes are illustrated in B) and D) (scale bar = 10 µm)

In order to examine the structure of the hybrid membranes, SEM images of both their surfaces and cross-sections were taken. The two individual layers (decellularized pericardium and polymer) appeared clearly distinct but mutually adherent: Fig. 7 A and Fig. 7 C depict the cross sections of the membranes where the decellularized pericardium lays over the polymeric sheet (Chronoflex AR and Chronoflex AR-LT, respectively). At higher magnification (Fig. 7B and D) it is possible to observe the presence of collagen bundles into the decellularized biological tissue. SEM images also revealed the characteristic patterns of the biological surface since collagen bundles are clearly visible: they are tidily aligned following a common direction.

**Fig. 8:**
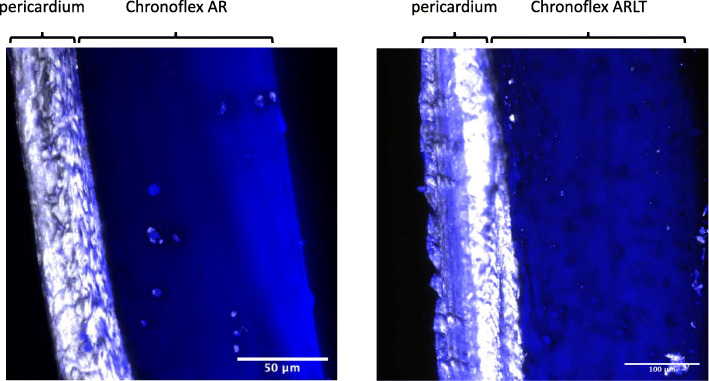
Two-photon microscopy. Cross-section images of DPP-AR (left) and DPP-ARLT (right) membranes

The interface between polymers and decellularized pericardia was also investigated by means of two-photon microscopy. Figure 8 depicts DPP in contact with the polymeric layers obtained with both Chronoflex AR and AR-LT. Polymeric and biological layers well adhered to each other and the interface was continuous. Moreover, the two-photon image confirmed that the polymer did not cross through the biological tissue.

## Discussion

The increasing exploitation of mechanical support devices for treating patients affected by refractory end-stage HF requires the development of hemocompatible materials. Anyway, hemocompatibility of blood-contacting surfaces is only one pace towards the hemocompatibility of the whole mechanical device, but it is of paramount importance in order to reduce the risk of adverse events (i.e., hemorrhages, hemolysis, thrombosis, and thromboembolism), which can severely compromise device’s functionality and patient’s safety.

In the present study, hybrid membranes were produced by coupling a commercial hemocompatible polycarbonate urethane (added and not added with silica particles) with decellularized porcine pericardium. Such hybrid materials are expected to combine the mechanical features of the synthetic polymer (in particular, fatigue resistance) and the hemocompatibility of the decellularized tissue. Unlike chemically treated tissues, the decellularized pericardium is prone to be repopulated by circulating cells. In this way, an endothelial layer will be generated in vivo over the blood-contacting side of the hybrid membrane [28]: a higher degree of hemocompatibility is thus obtained, resulting in a lower level of anticoagulation therapy for the benefit of the patient. Therefore, hybrid materials can be conveniently exploited for the construction of blood contacting devices, in particular for the pulsatile chambers of innovative VADs and TAHs. The advantages against the use of the GA-treated pericardium, as it has been adopted in the Carmat TAH, are also evident considering that GA can cause cytotoxic effects and tissue calcification and, eventually, device failure.

Biological analyses proved the effectiveness of TRICOL decellularization process: the DNA content has been reduced by more than 90 % and it is less than 50 ng per mg ECM dry weight in DPP. This is the threshold value below which adverse cells’ and host’s responses have been avoided in vivo, as suggested by Crapo et al. [29]. Effective DNA removal was also confirmed by Hoechst staining. Therefore, TRICOL procedure effectively decellularizes pericardium, without altering its histoarchitecture. Preserved collagen bundles organization, as it has been also displayed by microscopy, is responsible for the mechanical resistance of the membrane: it has to withstand stresses and strains resulting from the intended application in a ventricular chamber.

Before cytocompatibility and hemocompatibility assessments, which are under development, the hybrid membrane has been characterized with regard to its physico-chemical and mechanical features. Chemical composition of the composing layers has been ascertained by DSC, TGA and FTIR-ATR. Moreover, the coherence of the composing layers has been investigated: images taken by SEM and 2 photons microscopy ascertained that the interface developing upon contact between decellularized pericardium and synthetic polymer, is continuous and the layers are well adhering to each other. Polymeric and pericardial layers are perfectly stratified and polymers penetrates the biological tissue for a few microns: a good connection between the synthetic polymer and the biological tissue is then established. Additionally, the absence of any polymer trace on the pericardial side of the hybrid membrane has been proved by FTIR-ATR. Thus, decellularized pericardium will be in direct contact with blood assuring high hemocompatibility. Interestingly, SEM images give evidence of flattened collagen bundles, which lose their typical waviness. This effect can be due to the decellularization procedure that can alter the force of intra- and inter-molecular bonds.

Finally, biomechanical tests demonstrated the ability of the hybrid membranes to withstand the deformations that are foreseen during actuation of the intended ventricular chamber: the deformation level reached during the tests is much higher than that calculated during recently published simulations [30]. Briefly, three configurations of bellow-like pulsatile ventricular chambers have been compared by means of explicit dynamic computational analysis to identify maximum stress and strain values during actuation without any internal fluid (e.g., fluid–structure interactions have been neglected). During the simulated actuation of the best performing configuration, the maximum stress was lower than 2 MPa with a deformation of around 19 %. For the worst performing configuration, the strain raised up to 60 % but the stress remained under 3.5 MPa. In both cases, the registered stress and strain values were within the range of the experimentally measured ones. As depicted in Table 2, the proposed membranes can resist up to more than 70 % strain, with a UTS higher than 8 MPa. In line of principle, they are mechanically suitable for the intended application.

On the basis of the results obtained in terms of physicochemical and mechanical characterizations, it is not yet possible to select one polymer instead of the other: when coupled with DPP, both Chronoflex AR and AR-LT exhibit promising features. Empirically, the presence of silica microparticles into Chronoflex AR-LT makes the polymeric layer less tacky; on the other hand, Chronoflex AR is easier to be handled and molded. The ultimate selection of the most appropriate polymeric formulation has to be postponed when the results of other tests (fatigue resistance, cytocompatibility, hemocompatibility and calcification) will be available. Indeed, a preliminary investigation on the capacity of the membrane to evoke thrombin generation and platelet activation has been performed with promising results [31].

## Conclusions

The hybrid membranes fabricated by coupling decellularized porcine pericardium with a commercial polycarbonate urethane have been assessed in terms of composition, structure and mechanical resistance. As a result such hybrid membranes proved to be potentially suitable for the construction of the ventricular chambers of an innovative TAH.

For an exhaustive characterization of the membranes, further investigations will be necessary to assess fatigue resistance, cytocompatibility, hemocompatibility and calcification. Actually, cytocompatibility tests are in progress with HUVECs and MSCs following the UNI EN ISO 10993-5. The local ethical committee has already given the authorization for in vivo calcification tests on animal model (rodent), which has then been submitted to the Italian Ministry of Health.

## Data Availability

All data generated or analysed during this study are included in this published article.

## Abbreviations

BTT: Bridge To Transplantation
DPP: Decellularized Porcine Pericardium
DPP-AR: Hybrid membrane obtained by coupling Decellularized Porcine Pericardium and Chronoflex AR
DPP-ARLT: Hybrid membrane obtained by coupling Decellularized Porcine Pericardium and Chronoflex AR-LT
DMAc: dimethylacetamide
DT: Destination Therapy
EDTA: Ethylene Diamine Tetra Acetic Acid
FS: Failure Strain
GA: Glutaraldehyde
HF: Heart Failure
LLDPE: linear low density polyethylene
MDI: Methylene diisocyanate
NPP: Native Porcine Pericardium
OCT: Optimum Cutting Temperature
PCU: Polycarbonate urethane
PET: Polyethylene terephthalate
POSS: Polyhedral oligomericsilsesquioxanes
PNI: Pseudo-NeoIntima
SIBS: Poly(styrene-block-isobutylene-block-styrene)
TAH: Total Artificial Heart
UTS: Ultimate Tensile Strength
VAD: Ventricular Assist Device
